# SET-NUP214融合基因阳性血液恶性肿瘤24例临床特性分析

**DOI:** 10.3760/cma.j.issn.0253-2727.2021.06.004

**Published:** 2021-06

**Authors:** 淑敏 陈, 文杰 宋, 亚溱 秦, 峥 王, 辉 党, 岩 师, 琦 何, 倩 江, 浩 江, 晓军 黄, 悦云 赖

**Affiliations:** 北京大学人民医院，北京大学血液病研究所，国家血液系统疾病临床医学研究中心，造血干细胞移植治疗血液病北京市重点实验室 100044 Peking University People's Hospital, Peking University Institute of Hematology, National Clinical Research Center for Hematologic Disease, Beijing Key Laboratory of Hematopoietic Stem Cell Transplantation for Hematological Diseases, Beijing 100044, China

**Keywords:** 融合基因，SET-NUP214, 白血病, 异基因造血干细胞移植, SET-NUP214 fusion gene, Leukemia, Allogeneic hematopoietic stem cell transplantation

## Abstract

**目的:**

探讨SET-NUP214融合基因在血液恶性肿瘤中的表达，分析其相关的临床及生物学特征。

**方法:**

回顾性分析2012年1月至2018年12月北京大学人民医院诊断的24例SET-NUP214融合基因阳性血液恶性肿瘤患者的临床资料，并采用Kaplan-Meier法进行生存分析。

**结果:**

24例患者中，急性淋巴细胞白血病（ALL）15例（T-ALL 13例，B-ALL 2例）、急性髓系白血病（AML）7例，T/髓混合急性白血病2例。13例T-ALL患者免疫表型以CD3^+^CD2^−^为主要特征，73.3％的ALL患者伴有髓系标志表达，85.7％的AML患者表达CD7。24例患者诱导化疗完全缓解（CR）率91.7％。全部患者均接受异基因造血干细胞移植，中位随访24个月，AML和ALL的3年无复发生存（RFS）率分别为85.7％和33.3％，差异无统计学意义（*P*＝0.128）。比较13例SET-NUP214阳性与62例SET-NUP214阴性T-ALL患者的疗效，诱导化疗CR率分别为92.3％和93.5％（*P*＝0.445），诱导化疗4周CR率分别为69.2％和72.6％（*P*＝0.187），差异均无统计学意义。接受造血干细胞移植后，SET-NUP214阳性T-ALL患者的3年RFS率（38.5％）明显低于SET-NUP214阴性T-ALL患者（66.4％）（*P*＝0.028）。

**结论:**

SET-NUP214融合基因主要见于T细胞源性血液肿瘤，伴SET-NUP214融合基因T-ALL预后较差。

## Abstract (en)

**Objective:**

To investigate the expression of SET-NUP214 fusion gene in hematological malignancies and to analyze its related clinical biological characteristics.

**Methods:**

The clinical data of 24 patients with SET-NUP214 fusion gene-positive hematological malignancies were retrospectively analyzed, and the Kaplan-Meier method was used for survival analysis.

**Results:**

Among the 24 patients with SET-NUP214 fusion gene, 15 cases of acute lymphoblastic leukemia（ALL）（13 cases of T-ALL and 2 cases of B-ALL）, 7 cases of acute myeloid leukemia（AML）, and 2 cases of T/myeloid mixed acute leukemia have been identified. The immunophenotype of 13 cases of T-ALL was mainly characterized by CD3^+^CD2^−^, 73.3％ of ALL was characterized by myeloid marker expression, and 85.7％ of AML was characterized by CD7 expression. Complete remission （CR） was achieved in 22 patients （91.7％）after induction chemotherapy. All 24 patients received allogeneic hematopoietic stem cell transplantation（HSCT）. With a median follow-up of 24 months, the 3-year relapse free survival（RFS）of AML and ALL was 85.7％ and 33.3％, respectively（*P*＝0.128）. Comparing 13 cases of SET-NUP214-positive and 62 cases of SET-NUP214-negative T-ALL, the CR rates of induction chemotherapy were 92.3％ and 93.5％（*P*＝0.445）, and the 4-week CR rates of induction chemotherapy were 69.2％ and 72.6％, respectively（*P*＝0.187）; the differences were not statistically significant. After HSCT, the 3-year RFS of SET-NUP214^+^ T-ALL and SET-NUP214^−^ T-ALL was 38.5％ and 66.4％, respectively（*P*＝0.028）, and the difference was statistically significant.

**Conclusion:**

The SET-NUP214 fusion gene is mainly detected in T cell-derived hematological malignancies, and the prognosis of SET-NUP214 positive T-ALL is relatively poor.

SET-NUP214融合基因由染色体平衡易位t（9;9）（q34;q34）或9号染色体长臂3区4带隐匿性缺失del（9）（q34.11q34.13）形成，首次报道于一例急性未分化白血病患者[1]。SET-NUP214融合基因主要见于急性T淋巴细胞白血病（T-ALL），在T-ALL中发生率为3.0％～10.3％，在急性髓系白血病（AML）和急性B淋巴细胞白血病（B-ALL）中亦有报道[2]–[4]。目前认为具有SET-NUP214基因重排的血液恶性肿瘤患者预后较差，由于文献报道较少，对于此类疾病的特征尚缺乏较为一致的认识。本研究通过总结我院收治的24例SET-NUP214融合基因阳性血液恶性肿瘤患者的临床资料，以期初步探讨SET-NUP214基因重排在恶性血液病中的分布情况及其相关的临床生物学特性，提高对此类疾病的认识水平。

## 病例与方法

一、病例资料

回顾性分析2012年1月至2018年12月在我院诊断的24例SET-NUP214融合基因阳性血液恶性肿瘤患者的临床资料。患者采用骨髓细胞形态学、免疫学、细胞遗传学和分子生物学（MICM）诊断模式，疾病分型参照WHO造血系统和淋巴组织恶性肿瘤分型标准（2017年）。同时回顾性分析本单位同期62例18～60岁有完整随访资料的SET-NUP214融合基因阴性T-ALL患者的临床资料。

二、方法

1. 基因检测：采用实时荧光定量PCR法检测SET-NUP214等白血病相关融合基因以及WT1、PRAME、NPM1等基因，以abl作为内参基因，同时扩增含有abl基因的标准质粒，制备标准曲线，并计算每份标本被检测目的基因和abl基因的拷贝数，结果以两者拷贝数比值的百分数来表示，具体步骤参照文献[5]。T细胞受体基因（TCR）、IKZF、HOXA等基因检测采用DNA定性或定量PCR法。FLT3-ITD采用PCR结合毛细管电泳法检测。

2. 染色体核型分析和荧光原位杂交（FISH）检测：常规采用24 h短期培养和G显带法进行核型分析，染色体标本制备参照本单位细胞遗传学实验室常规方法。核型命名参照《人类细胞遗传学国际命名体制（ISCN2016）》进行描述。每次核型分析尽可能分析20个中期分裂细胞且至少经过2位医师共同鉴定。患者初诊时的骨髓染色体标本经常规核型分析后剩余的细胞悬液制备成FISH检测标本，按照FISH操作步骤进行FISH检测[6]。LSP ABL1双色分离探针购自美国Cytotest公司，LSP ABL1 5′端标记为红色，3′端标记为绿色，均用Olympus BX51荧光显微镜在DAPI/FITC/TexasRed三色滤光镜激发下观察200个间期细胞荧光杂交信号。

3. 流式细胞术检测细胞免疫表型：采用八色流式细胞术检测，参照本单位常规方法完成[7]，并根据每例患者特征性免疫表型进行治疗后的免疫残留分析。所用抗原包括HLA-DR、TdT、CD34、CD38、CD45、CD1a、CD2、CD3、CD4、CD5、CD8、CD10、CD19、CD20、CD22、CD79a、cIgM、mIgM、FMC7、MPO、CD11b、CD13、CD14、CD15、CD33、CD64等。

4. 治疗方案及疗效评估：24例SET-NUP214融合基因阳性患者和62例SET-NUP214融合基因阴性患者均在我院诊断治疗并评估疗效，诱导化疗4周后进行骨髓穿刺评估疗效，获得完全缓解（CR）的患者给予巩固治疗，未获得CR的患者进行再次诱导化疗。ALL患者首选CODP（环磷酰胺+长春新碱+柔红霉素+地塞米松）或CODPL（环磷酰胺+长春新碱+柔红霉素+地塞米松+左旋门冬酰胺酶）方案诱导化疗，巩固方案主要包括CODP或Hyper CVAD-A方案（环磷酰胺+吡柔比星+长春新碱+地塞米松）、Hyper CVAD-B方案（甲氨蝶呤+阿糖胞苷），其治疗方案参考《中国成人急性淋巴细胞白血病诊断与治疗指南（2016年版）》[8]。本研究15例SET-NUP214融合基因阳性ALL患者中14例采用CODP/CODPL+ Hyper CVAD-A/B方案，1例采用VDLD（长春地辛+柔红霉素+培门冬酰胺酶+地塞米松）方案。62例SET-NUP214阴性T-ALL患者诱导和巩固治疗方案均采用CODP/CODPL+ HyperCVAD-A/B方案。

AML患者首选IA（去甲氧柔红霉素+阿糖胞苷）或DA（柔红霉素+阿糖胞苷）或MA（米托蒽醌+阿糖胞苷）方案诱导化疗，巩固治疗方案主要包括IA或中大剂量阿糖胞苷方案等；本研究7例AML患者均采用IA或MA方案诱导化疗。2例T/髓混合急性白血病患者的化疗方案则选择兼顾髓系和淋系白血病的方案CODLP或VPIA（长春新碱+泼尼松+去甲氧柔红霉素+阿糖胞苷）。CR定义为骨髓中原始细胞比例<5％，无髓外白血病症状。复发指CR后外周血再次出现白血病细胞或骨髓中原始细胞≥5％（除外巩固化疗后骨髓再生等其他原因）或髓外出现白血病细胞浸润。

5. 异基因造血干细胞移植（allo-HSCT）方案：HLA半相合移植受者预处理方案均采用本科室常规改良的BuCy方案联合应用抗胸腺细胞球蛋白，HLA全相合移植受者预处理方案为改良的BuCy方案[9]。移植物抗宿主病（GVHD）的预防均采用本科室常规方案：环孢素A+霉酚酸酯+短程甲氨蝶呤[10]。

6. 随访：采用查阅病历或电话的方式进行随访，随访截止时间为2020年12月31日。总生存（OS）期定义为从确诊至任何原因引起死亡或者末次随访的时间。无复发生存（RFS）期定义为从第1次获得CR（CR_1_）至疾病复发或死亡的时间。

三、统计学处理

应用SPSS22.0软件进行统计学分析，计量资料以中位数和范围表示。生存分析采用Kaplan-Meier方法，生存率的比较采用Log-rank检验。*P*<0.05为差异有统计学意义。

## 结果

一、患者一般情况

24 例SET-NUP214融合基因阳性患者中，男19例，女5例，中位年龄30（12～58）岁，根据WHO分型，诊断ALL 15例（T-ALL 13例，B-ALL 2例），AML 7例（M_0_ 4例，M_2_ 2例，M_5_ 1例），T/髓混合急性白血病2例。

二、实验室检查特征

1. 细胞遗传学特征：24例患者均进行了染色体G显带核型分析，其中正常核型12例，异常核型12例。12例异常核型中6例可见t（9;9）（q34;q34），其中4例伴有附加异常（表1）。有4例患者利用ABL1双色分离探针进行了FISH检测，3例主要异常信号类型为1绿1黄（1G1F）（异常细胞比例分别为86.5％、83.0％和79.0％），提示ABL1基因5′端缺失，1例异常信号主要包括1G1F（10％）和1G2F（9％），后者提示具有ABL1基因5′端缺失的同时伴有ABL1基因的扩增。

**表1:** 24例SET-NUP214阳性血液肿瘤患者临床资料

例号	性别	年龄(岁)	诊断	染色体核型	基因	CR情况	移植方案	复发状态	无复发生存(月)	转归	OS(月)
1	男	20	AML-M_2_	46,XY,t(9;9)(q34;q34)[3]/46,XY[18]	SET-NUP214:172.6%, WT1:53.5%	1个疗程CR	全相合	未复发	33	存活	34
2	男	58	T-ALL	46,XY,der(11)add(11)(p15)del(11)(q21)[1]/47,idem,+21[1]/48,idem,+21,+22[2]/46,XY,t(9;9)(q34;q34)[2]/46,XY[14]	SET-NUP214:263.0%	1个疗程CR	全相合	未复发	34	存活	35
3	女	27	T-ALL	44,XX,-8,t(9;9)(q34;q34),-11[1]	SET-NUP214:598.0%, WT1:38.5%,HOX11:2.0%, TCRδ(+),P53(+)	1个疗程CR	半相合	复发	6	死亡	24
4	男	37	T-ALL	46,XY,t(9;9)(q34;q34)[4]/46,XY[16]	SET-NUP214:1098.0%, WT1:38.3%,TCRδ(+)	1个疗程CR	全相合	未复发	58	存活	59
5	男	32	AML-M_0_	46,XY,t(9;9)(q34;q34)[5]/46,idem,i(17)(q10)[3]/46,XY[10]	SET-NUP214:242.0%, WT1:51.6%	1个疗程CR	全相合	未复发	35	存活	40
6	男	27	T-ALL	46,XY,add(11)(p13)[4]/46,idem,+12,-18[1]	SET-NUP214:743.0%, WT1(+),TCRδ(+)	1个疗程CR	半相合	复发	5	死亡	26
7	女	16	T-ALL	46,XX[17]	SET-NUP214:542.7%	2个疗程CR	全相合	未复发	38	存活	41
8	男	18	B-ALL	56,XY,+6,+8,+12,+13,+15,+19,+20,+21, +21,+mar1[1]/45-49,XY,+12,+15,+16, i(17)(q10),+21,+22,+mar2[cp5]/46,XY[4]	SET-NUP214:567.0%, WT1:46.5%	NR	半相合	未复发	3	死亡	9
9	男	36	T-ALL	46,XY,del(4)(q31),del(6)(q21),add(8)(q24),add(11)(p13),del(14)(q22),i(17)(q10)[5]/46,XY[15]	SET-NUP214:726.0%, TCRδ(+)	1个疗程CR	全相合	复发	14	死亡	15
10	男	26	AML-M_0_	43,XY,-2,-2,-8[2]	SET-NUP214:131.0%, WT1:15.0%	1个疗程CR	半相合	复发	55	存活	90
11	女	22	T/髓混合细胞白血病	46,XX[20]	SET-NUP214:673.0%, WT1: 17.4%,TCRγ(+),TCRδ(+)	1个疗程CR	半相合	未复发	41	存活	42
12	男	12	AML-M_5_	46,XY[20]	SET-NUP214:500.3%	1个疗程CR	半相合	未复发	31	存活	32
13	男	22	B-ALL	92,XXYY,i(17)(q10)×2[3]/46,XY[20]	SET-NUP214:90.6%, WT1:6.3%,TCRδ(+)	1个疗程CR	半相合	复发	13	死亡	15
14	女	46	AML-M_0_	46,XX[9]	SET-NUP214:55.6%, WT1:98.6%	1个疗程CR	全相合	未复发	37	存活	41
15	男	38	AML-M_2_	46,XY[20]	SET-NUP214:62.0%	1个疗程CR	全相合	复发	39	死亡	45
16	男	40	T-ALL	46,XY[20]	SET-NUP214:643.5%, WT1:111.0%	1个疗程CR	全相合	复发	11	死亡	18
17	男	41	T-ALL	46,XY[4]	SET-NUP214:73.9%, WT1:16.1%	NR	半相合	未复发	22	死亡	22
18	女	34	T-ALL	46,XX[17]	SET-NUP214:128.4%, WT1:2.7%,TCRδ(+)	1个疗程CR	半相合	未复发	50	存活	51
19	男	15	T-ALL	46,XY[5]	SET-NUP214:512.6%	2个疗程CR	半相合	复发	9	死亡	12
20	男	50	AML-M_0_	46,XY[6]	SET-NUP214:1040.3%, WT1:1.0%	2个疗程CR	全相合	复发	17	死亡	25
21	男	12	T-ALL	46,XY[20]	SET-NUP214:118.7%	2个疗程CR	半相合	复发	17	死亡	24
22	男	34	T/髓混合细胞白血病	46,XY[20]	SET-NUP214:533.1%, WT1:51.0%	2个疗程CR	半相合	未复发	22	存活	24
23	男	42	T-ALL	46,XY,+Y,-9[1]/46,XY[9]	SET-NUP214:611.5%, WT1:43.8%,TCRδ(+)	1个疗程CR	半相合	未复发	26	存活	29
24	男	36	T-ALL	46,XY,t(9;9)(q34;q34)[2]//46,idem, add(17)(p13)[2]/46,XY[2]	SET-NUP214:1462.9%, WT1:120.3%,TCRδ(+)	1个疗程CR	半相合	复发	10	存活	14

注：AML：急性髓系白血病；T-ALL：急性T淋巴细胞白血病；B-ALL：急性B淋巴细胞白血病；CR：完全缓解；NR：未缓解；OS：总生存

2. 分子生物学特征：24例患者起病时SET-NUP214融合基因水平中位值为598.0％（55.6％～1462.0％）；24例患者初诊时均进行了WT1基因检测，17例表达升高，WT1水平中位值为38.4％（1.0％～120.3％）。13例T-ALL患者均检测TCR基因（包括TCRδ和TCRγ），其中7例TCRδ阳性。7例ALL患者（T-ALL 5例，B-ALL 2例）检测了IKZF基因，均为阴性。

3. 免疫分型特征：15例ALL（13例T-ALL和2例B-ALL）患者均表达异常淋系标志（13例表达幼稚T淋巴细胞标志，2例表达幼稚B淋巴细胞标志），其中9例（69.2％）T-ALL和2例（100％）B-ALL伴有髓系标志表达（主要包括CD13、CD33、CD15和CD117等），13例T-ALL患者免疫表型以CD3^+^CD2^−^为主要特征，6例（46.2％）CD5阳性。7例AML患者均表达异常髓系标志，其中6例（85.7％）具有CD7表达。

三、治疗情况及治疗反应

经过诱导化疗，24例患者中22例（91.7％）获得CR。15例ALL患者的诱导化疗CR率为86.7％，其中10例（66.7％）诱导化疗1个疗程（4周）达CR，3例（20.0％）经2～3个疗程诱导化疗达到CR，2例（13.3％）经化疗始终未能获得CR。7例AML患者中6例（85.7％）1个疗程达CR，1例（14.3％）2个疗程达CR。2例T/髓混合急性白血病患者1例诱导化疗1个疗程即获得CR，1例2个疗程达CR。24例患者均进行了allo-HSCT，包括14例HLA半相合移植和10例HLA全相合移植，22例获得CR的患者在接受2～6个疗程巩固化疗后接受allo-HSCT，2例未缓解（NR）患者分别在接受2和3个疗程诱导化疗后进行了移植。

四、复发和生存分析

1. 复发情况：24例患者移植后11例患者血液学复发，包括ALL 8例，AML 3例。8例ALL患者分别于移植后2.5、4、5.5、8、12、16、31、32个月复发，3例AML患者分别于移植后7、21、37个月复发。11例患者死亡，其中9例（T-ALL 6例，B-ALL 1例，AML 2例）死于复发，死亡时间分别为移植后2.5、9、10、13、15、17、19、31、41个月；2例（T-ALL 1例，B-ALL 1例）死于GVHD，此2例患者移植前均NR，移植后分别存活3和16个月。详见表1。

2. 生存分析：24例患者中位随访时间为24（9～94）个月，中位OS期为45个月，3年OS率为56.1％。中位RFS期为39个月，3年RFS率为54.2％。按不同疾病分组，AML和ALL组的3年OS率分别为85.7％和36.1％（*P*＝0.123），3年RFS率分别为85.7％和33.3％（*P*＝0.128），差异均无统计学意义。

五、连续动态监测SET-NUP214融合基因转录本水平在预测复发中的作用

17例患者诱导化疗1个疗程即达CR，CR时SET-NUP214融合基因水平中位值为2.6％（0～102.0％），其中11例下降幅度≥2个数量级，6例下降幅度≤1个数量级。5例患者2个疗程后达到CR，CR时SET-NUP214融合基因水平中位值为0.4％（0～36.3％），下降幅度均≤1个数量级。2例患者NR，1例患者化疗后融合基因水平最低为5.1％，另1例移植前融合基因水平最低为14.6％，此两例患者移植后均获得CR，融合基因水平最低为0。

CR后随访期间，13例未复发患者SET-NUP214融合基因表达水平均持续低于0.001％，直至观察终点；11例复发患者CR时SET-NUP214水平最低均降至0，CR后连续监测SET-NUP214融合基因水平，从>0.001％开始到形态学复发中位时间为5（2～12）个月，复发时SET-NUP21融合基因中位值58.7％（2.1％～237.7％）。

六、SET-NUP214^+^ T-ALL和SET-NUP214^−^ T-ALL疗效比较

比较本单位13例SET-NUP214^+^ T-ALL与62例SET-NUP214^−^ T-ALL患者的疗效，诱导化疗CR率分别为92.3％和93.5％（*P*＝0.445），诱导化疗4周CR率分别为69.2％和72.6％（*P*＝0.187），两组差异无统计学意义。比较接受造血干细胞移植的13例SET-NUP214^+^ T-ALL与35例SET-NUP214^−^ T-ALL患者生存数据，3年OS率分别为42.0％和74.4％，差异无统计学意义（*P*＝0.104）；3年RFS率分别为38.5％和66.4％（*P*＝0.028）（图1），SET-NUP214^+^ T-ALL患者3年RFS率显著低于SET-NUP214^−^ T-ALL患者。

**图1:**
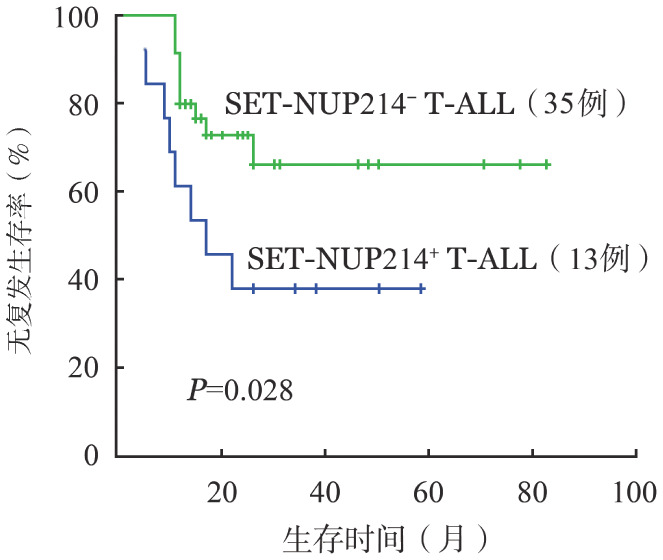
接受移植的SET-NUP214^+^与SET-NUP214^−^急性T淋巴细胞白血病（T-ALL）患者无复发生存的比较

## 讨论

SET-NUP214由隐匿的t（9;9）（q34.11;q34.13）或del（9）（q34.11q34.13）形成，是血液肿瘤中一种少见的融合基因，常规的染色体核型分析难以发现形成此融合基因的隐匿性染色体异常，需要依靠分子生物学方法如RT-PCR或基因测序证实。目前SET-NUP214阳性血液肿瘤国内外相关文献报道不足50例，且主要见于T-ALL，在AML和B-ALL中SET-NUP214融合基因较为罕见，分别仅见5例和2例报道[11]–[13]。本研究24例SET-NUP214融合基因阳性患者中，T-ALL 13例（54.2％）、AML 7例（29.2％）、B-ALL2例（8.3％），此外还有2例T/髓混合急性白血病。从疾病分布表明，SET-NUP214融合基因主要见于T细胞源性肿瘤，但并非系列特异性标志。此外，本组24例患者仅6例（25％）G显带提示具有t（9;9）（q34;q34）异常，即使用ABL1双色分离探针进行FISH检测，只能显示具有ABL1基因的部分缺失，无法区分t（9;9）平衡易位和del（9）（q34.11q34.13），亦无法确定是否为SET-NUP214融合。上述结果印证了常规染色体核型分析和FISH检测对此类异常检出的局限性。

SET-NUP214融合基因阳性血液恶性肿瘤的实验室特性与疾病分型有关。Ben等[14]报道11例SET-NUP214阳性T-ALL，免疫表型以CD5^+^CD2^−^为主要特征，TCR基因主要为TCRγ和（或）TCRδ，其中9例（82％）表达髓系标志。文献报道伴CD13^+^、CD33^+^等髓系标志是SET-NUP214阳性T-ALL的重要特征[4],[15]–[17]。本研究13例SET-NUP214^+^ T-ALL患者中，9例（69.2％）表达髓系标志，与Ben等[14]报道类似。Ben等通过筛查22例HOXA基因高表达的AML患者，发现1例CD7^+^且同时具有TCRγ和TCRδ重排的SET-NUP214^+^AML-M_0_患者。董晓燕等[18]报道的2例SET-NUP214^+^AML均表达CD7，提示CD7^+^可能是SET-NUP214^+^AML的免疫表型特征之一。CD7跨膜糖蛋白正常情况下主要在T和NK细胞及其前体细胞中表达，文献报道约30％的AML可表达CD7且与疾病进展和化疗耐药相关[19]–[20]。本组资料7例AML患者中6例（85.7％）表达CD7，本研究进一步证实CD7在绝大部分SET-NUP214^+^ AML中高表达。Gomes-Silva等[21]利用CD7在部分AML原始细胞中高表达而在正常髓系细胞和红系细胞中不表达的特性在小鼠移植模型中证实，针对CD7的CAR-T细胞治疗能有效抑制白血病而延长生存。本研究结果为AML的CAR-T细胞领域研究进一步提供理论依据。

SET-NUP214融合基因阳性血液恶性肿瘤的预后具有较大异质性，多数研究提示SET-NUP214预后不良，移植可改善患者生存。Ben等[14]分析196例成人T-ALL，其中11例（6％）具有SET-NUP214融合基因，与SET-NUP214阴性患者比较，SET-NUP214阳性T-ALL患者对激素（91％对44％，*P*＝0.003）和化疗（100％对44％，*P*<0.001）更易发生耐药，早期治疗反应差，但移植后3年OS和EFS率与SET-NUP214阴性患者差异无统计学意义（73％对68％，*P*＝0.86；45％对59％，*P*＝0.52），提示移植可改善其预后。在Gorello等[22]的研究中，6例SET-NUP214阳性T-ALL患者中4例在诊断后12～24个月内死于疾病难治或复发。戴海萍等[23]也观察到类似情况，6例阳性患者中，4例在病程不同阶段出现疾病复发，3例死亡，中位RFS时间为7.8个月。董晓燕等[18]分析4例SET-NUP214融合基因阳性急性白血病患者的临床特征，提示SET-NUP214阳性患者整体预后差，对文献报道的23例具有详细生存资料的SET-NUP214阳性患者进行生存分析发现化疗组（7例）2年OS率显著低于移植组（16例）［（33.9±19.2）％对（86.2±9.1）％，*P*＝0.012］，提示移植显著改善此类患者生存。本研究报道的7例SET-NUP214阳性AML患者诱导化疗CR率为100％，移植后3年RFS率为85.7％，与本中心既往报道的AML生存接近[24]。我们将13例SET-NUP214阳性T-ALL与本单位SET-NUP214阴性T-ALL进行比较，两组化疗CR率差异无统计学意义，接受移植后3年OS率分别为42.0％和74.4％（*P*＝0.104），3年RFS率分别为38.5％和66.4％（*P*＝0.028），此结果提示，SET-NUP214阳性T-ALL患者即使接受造血干细胞移植，其3年RFS率亦显著低于SET-NUP214阴性T-ALL患者，预后较差。此外，值得提出的是通过对SET-NUP214基因转录本水平的连续监测，我们发现SET-NUP214融合基因表达水平持续低于0.001％，是患者获得长期RFS的预示标志。基因水平由0到低拷贝阳性，从>0.001％开始到形态学复发中位时间为5个月，提示SET-NUP214融合基因可作为有效的微小残留病监测指标，连续监测能提早预测疾病复发。

本研究通过对24例SET-NUP214融合基因阳性血液恶性肿瘤患者的资料进行分析，证明SET-NUP214作为一种罕见的融合基因，多发生于T细胞源性血液肿瘤，但无系列特异性。移植后SET-NUP214阳性T-ALL 3年RFS率显著低于SET-NUP214阴性T-ALL，预后较差。连续监测SET-NUP214融合基因转录本水平可更早地预测疾病的复发，为临床早期干预提供依据。
